# Determining the prevalence of palliative needs and exploring screening accuracy of depression and anxiety items of the integrated palliative care outcome scale – a multi-centre study

**DOI:** 10.1186/s12904-020-00571-8

**Published:** 2020-05-14

**Authors:** Bárbara Antunes, Pedro Pereira Rodrigues, Irene J. Higginson, Pedro Lopes Ferreira

**Affiliations:** 1 Primary Care Unit, Department of Public Health and Primary Care, University of Cambridge, University Forvie Site, Robinson Way, Cambridge Biomedical Campus, Cambridge, CB2 0SR UK; 2 Centre for Health Technology and Services Research, Alameda Prof. Hernâni Monteiro, 4200-319 Porto, Portugal; 3 King’s College London, Cicely Saunders Institute, Bessemer Road, London, SE5 9PJ UK; 4 Centre for Health Studies and Research, Av. Dias da Silva, 165, 3000-512 Coimbra, Portugal

**Keywords:** Palliative care, Screening accuracy, Depression, Anxiety, Patient centred outcome measures, Outcome measurement, End of life care, Suffering

## Abstract

**Background:**

patients with palliative needs often experience high symptom burden which causes suffering to themselves and their families. Depression and psychological distress should not be considered a “normal event” in advanced disease patients and should be screened, diagnosed, acted on and followed-up. Psychological distress has been associated with greater physical symptom severity, suffering, and mortality in cancer patients. A holistic, but short measure should be used for physical and non-physical needs assessment. The Integrated Palliative care Outcome Scale is one such measure. This work aims to determine palliative needs of patients and explore screening accuracy of two items pertaining to psychological needs.

**Methods:**

multi-centred observational study using convenience sampling. Data were collected in 9 Portuguese centres. Inclusion criteria: ≥18 years, mentally fit to give consent, diagnosed with an incurable, potentially life-threatening illness. Exclusion criteria: patient in distress (“unable to converse for a period of time”), cognitively impaired. Descriptive statistics used for demographics. Receiving Operator Characteristics curves and Area Under the Curve for anxiety and depression discriminant properties against the Hospital Anxiety and Depression Scale.

**Results:**

1703 individuals were screened between July 1st, 2015 and February 2016. A total of 135 (7.9%) were included. Main reason for exclusion was being healthy (75.2%). The primary care centre screened most individuals, as they have the highest rates of daily patients and the majority are healthy. Mean age is 66.8 years (SD 12.7), 58 (43%) are female. Most patients had a cancer diagnosis 109 (80.7%). Items scoring highest (=4) were: family or friends anxious or worried (36.3%); feeling anxious or worried about illness (13.3%); feeling depressed (9.6%). Using a cut-off score of 2/3, Area Under the Curve for depression and anxiety items were above 70%.

**Conclusions:**

main palliative needs were psychological, family related and spiritual. This suggests that clinical teams may better manage physical issues and there is room for improvement regarding non-physical needs. Using the Integrated Palliative care Outcome Scale systematically could aid clinical teams screening patients for distressing needs and track their progress in assisting patients and families with those issues.

## Background

Palliative care is a holistic approach of care, which can be integrated early in the disease trajectory, alongside active, curative treatment, [1] and aims to alleviate physical and non-physical symptoms of patients and their families [2–7]. Though physical symptoms may be more easily identified by healthcare professionals, patients and carers, non-physical symptoms can equally disrupt the patient and family’s quality of life and cause suffering [8]. Regarding effectiveness of psychological interventions to improve quality of life in people with long term conditions, results from a rapid systematic review show that there was a significant improvement in at least one quality of life outcome post-intervention and maintained at follow-up [9]. In another study, Ann-Yi S and colleagues report that 24% of palliative care in-patients and 19% of palliative care outpatients in a major cancer centre benefited from psychology services [8]. Also, psychological distress has been associated with greater physical symptom severity, suffering, and mortality in cancer patients [10]. Thus, these symptoms and needs should be properly assessed with validated outcome measures, and, the intervention(s) to solve or manage them should be selected accordingly [11, 12]. Depression and psychological distress are two examples of such needs, which should not be considered a “normal event” in an advanced disease trajectory [13]. Rather, they should be screened, diagnosed, acted on and followed-up by appropriate support services and specialised healthcare professionals, whether by pharmacologic treatment, psychological treatment, psychiatric treatment or a combination of those [13]. Patient centred outcome measures should be the first choice to measure subjective symptoms and needs, given that the patient is the best person to assess how the symptoms bother them. If the patient is cognitively impaired, a proxy version of the measure of choice can be used [14–18]. These measures are short, but multidimensional and some items may be used to screen for certain palliative needs, common in this population [19].

The aim of this study is to determine the main palliative care needs of patients being treated in portuguese health care services and to explore screening properties of two items pertaining to psychological needs, using the Integrated Palliative care Outcome Scale (IPOS) [20, 21]. We hypothesized that 1) anxiety and depression items will score highest among the non physical symptoms and 2) the area under the curve (AUC) to be > 0.7 for Items 3 (anxiety) and 5 (depression) in relation to the Portuguese Hospital Anxiety and Depression Scale (HADS) [22] Anxiety subscale score and the HADS-Depression subscale total scores, respectively.

## Methods

### Patients and settings

This was a multi-centred observational study. Data were collected in nine portuguese centres spread out from north to south and rural to urban locations to maximise generalizability using convenience sampling. There were seven hospital based palliative care services, one oncology service and one primary care facility (health centre). All patients attending the participant services were screened for eligibility by the participating healthcare professionals. Inclusion criteria were as follows: ≥18 years, mentally fit to give consent judged as such by the participating healthcare professional, diagnosed with an incurable, potentially life-threatening illness, read, write and understand Portuguese. Exclusion criteria were: patient in distress (unable to maintain a conversation during a period of time) with uncontrolled physical or emotional symptoms, and/or cognitively impaired, judged as such by the participating healthcare professional. A standard operating procedures manual was developed and distributed to all centres in the person of the facilitator/champion leading the study locally. The detailed protocol has been published elsewhere [18].

### Measures

The Portuguese version of the IPOS reported by the patient, previously developed, was used. The original measure has been culturally adapted and validated to European Portuguese [20, 21]. The protocol of the latter study has been published elsewhere [18] which contains the full questionnaire in the appendix. Next, we present a summary of main procedures and results. Two native Portuguese speaking translators, one clinical and one non-clinical independently created two Portuguese versions. A consensus Portuguese version was developed by two native Portuguese speaking independent reviewers blind to the original IPOS. This consensus version was sent to two other independent native Portuguese speaking translators, also blind to the original English IPOS, who back translated it into English. A second Portuguese consensus version was developed by the same reviewers. Three clinical revisions were performed by one specialist palliative care doctor, one specialist palliative care nurse and one non-clinical researcher – all native Portuguese. A final Portuguese version was created. There were grammatical and content differences in the first translation stage, in the items/questions text as well as in the response categories. These were resolved by discussion by both reviewers. There were also differences in the backward translation, namely verb tenses and the use of synonyms rather than the direct translation of words. These were resolved by discussion by the same reviewers. The clinical revisions flagged differences in verb tenses in three items. Those were discussed, and changes were made to create the final version. A Portuguese version of the IPOS was developed. Next, psychometric characteristics were assessed, namely, Internal consistency (excluding open questions, which are free text data) Cronbach’s alpha varied between 0.68 and 0.72, reliability between patients and healthcare professionals scores was assessed by intraclass correlation which was higher for mobility (ICC = 0.726 and lowest for practical problems (ICC = 0.088). Regarding construct validity, Items with similar constructs showed convergent validity and items with different constructs showed divergent validity. Spearman’s Rho varied between .390 and .631 with *p* ≤ .000. The measure also displayed good sensitivity to change, as Wilcoxon ranked test showed significant statistically differences between T1 and T2 in three symptoms. IPOS It is a brief, 19-item, multidimensional scale that captures core concerns in palliative care. The first item is an open question on the three main problems or worries the respondent might have had in the past week (results of this item are not reported in the present study given that data are free text); items 2 to 9 are set on a 5 point Likert scale based on descriptors (zero – not at all, 1 – slightly, 2 – moderately, 3 – severely, 4 – overwhelmingly), item two is a list of 10 of the most common physical symptoms in a palliative population, with the possibility of adding up to three more symptoms which are not present in the list; item 3 pertains to anxiety, item 4 asks about family/friends worry, item 5 is on depression; item 6 is about being at peace; item 7 relates to sharing feelings with significant people; item 8 is about information needs and item 9 concerns practical problems related to their illness. In the patient version (as opposed to the healthcare professional version), the questionnaire has an extra item asking if the respondent filled the questionnaire alone or with help. At the very end, there is a footnote to trigger the patient to talk to their healthcare professional, if they feel they are worried about any of the issues raised by the items in the questionnaire. This feature allows for real time clinical utility of the measure.

The Portuguese Hospital Anxiety and Depression Scale (HADS) is a 14 item screening measure for anxiety and depression states. The two subscales are comprised of 7 items each, scored separately, with descriptive answers based in a 4 point Likert scale. The authors propose a cut-off threshold of 11 for depression and anxiety. The authors conclude that the Portuguese HADS is a reliable and valid measure for assessing anxiety and depression in different medical settings and disease populations [22].

### Analysis

After checking data quality and performing Little’s Missing Completely At Random (MCAR) test to check if data were missing at random, descriptive statistics were used to examine the distribution of demographic and clinical variables of interest. To determine accuracy of the items under study, we previously tested all possible cut-offs (results not presented) and decided to use the most appropriate, a cut-off score of 2/3. Then we compared all 5 psychological, emotional and spiritual needs items (IPOS items 3 to 7) against the Portuguese HADS. Receiver operating characteristic curves (ROC) were used to determine the 2 items displaying the highest AUC and assess discriminant properties, namely, sensitivity and specificity of the cut-off 2/3 for both items against cut-off 10/11 of the HADS Anxiety subscale and the HADS Depression subscale respectively. Positive (PPV) and negative predictive values (NPV), false negative (FNR) and false positive rates (FPR) and positive and negative likelihood ratios weighted by prevalence were also computed for both items. For sensitivity, specificity, PPV, and NPV we considered 70% or above values to be acceptable and 80% or above to be high. For FPR and FNR of 30% or less, we considered them to be low. 95% confidence intervals were used. No sample size calculation was performed given that the only study found in the literature used the Palliative care Outcome Scale questionnaire (not the IPOS) and the HADS to screen for depression and anxiety was a secondary analysis of several independent datasets.

### Ethics procedures

Ethical approval was granted from all relevant Research Ethics Committees and was in accordance with the 1964 Helsinki declaration and its later amendments or comparable ethical Standard. All participants gave informed signed consent. SPSS, version 22 (SPSS/IBM Corp., Armonk, NY) software was used.

## Results

A total of 1703 individuals were screened between July 1st, 2015 and February 2016, the majority of which in a health centre, a primary health care facility. There were 18 (1.1%) patients eligible for the study who declined participation and 140 (8.2%) were excluded. A total of 135 (7.9%) patients were included (See Table 1).

**Table 1:** Patients screened and included in the study by participating centre

Participating services	Screened N (%)	Included N (%)
Oncology Hospital service	78 (4.6)	25 (18.5)
Palliative care service 1 north	96 (5.6)	9 (6.7)
Palliative care service 2 north	28 (1.6)	17 (12.6)
Palliative care service 3 south	18 (1.1)	1 (0.7)
Palliative care service 4 north	77 (4.5)	24 (17.8)
Palliative care service 5 south	64 (3.8)	17 (12.6)
Primary care centre 1 south	119 (7.0)	3 (2.2)
Primary care centre 2 south	1177 (69.1)	25 (18.5)
Palliative care service 6 centre	46 (2.7)	14 (10.4)
Total	1703 (100)	135 (100)

Main reason for exclusion was being healthy (75.2%). This is expected given that the primary care centre screened most individuals, as they have the highest rates of daily patients and most of them are healthy. Mean age is 66.8 years (SD 12.7), 58 (43%) are female, 74 (54.8%) have up to 4 years of formal education, 74 (54.8%) are from the Northern region. Most patients (*N* = 109, 80.7%) had a cancer diagnosis and came from the 7 hospital palliative care services (See Table 2).

**Table 2:** Demographic and clinical information of participants Demographic and clinical information N(%)

Mean Age in years (SD)		66.8 (12.7)
Gender Male		77 (57%)
Education (in years)	Reads and writes	5 (3.7)
	4 years	81 (60)
	6 years	20 (14.8)
	9 years	10 (7.4)
	10 years to college	19 (14)
Geographical region	North	74 (54.8)
	Centre	25 (18.5)
	South	36 (26.7)
Area	Urban	94 (69.6)
	Peri-urban	31 (23)
	Rural	10 (7.4)
Place of care	Primary care	28 (20.7%)
	hospital services	25 (18.5%)
	palliative care services	82 (60.7%)
Cancer Diagnosis		109 (80.7)
Phase of illness	Stable	64 (47.4)
	Unstable	28 (20.7)
	Deteriorating	43 (31.9)
	Terminal	
Surprise question (life expectancy)	> 1 year	37 (27.4)
	6 m to 1 year	45 (33.3)
	< 6 months	48 (35.6)

The main reasons for ineligibility and exclusion from the study are presented in Table 3. Most patients were approached to participate in the study whilst in external consultation, 98 (72.6%). About 31.1% were able to fill the questionnaires without help.

**Table 3:** Reasons for ineligibility and exclusion

	Reasons for ineligibility and exclusion	N (%)
Ineligible	< 18 years	91 (5.9)
	Does not understand Portuguese	1 (0.06)
	Cannot read or write	78 (5.0)
	Illness with possibility of cure	3 (0.2)
	Healthy	1165 (75.2)
	No reason stated	72 (4.6)
Excluded	Distress	52 (3.4)
	Cognitive deterioration	73 (4.7)
	No reason stated	15 (0.9)
Total		1550 (100)

Data were missing at random (Little’s MCAR test showed Chi-Square = 2452.946, DF = 2398, Sig. = .213). Missing data varied between 1 and 5% (rates < 1% are trivial, 1–5% are manageable, 5–15% require sophisticated statistical methods to handle, and > 15% may severely impact any form of interpretation.) [23]. As expected in palliative populations, most questionnaire items presented a non-parametric distribution, so the imputation of the median was used to handle missing data.

### Prevalence of needs

**Fig. 1:**
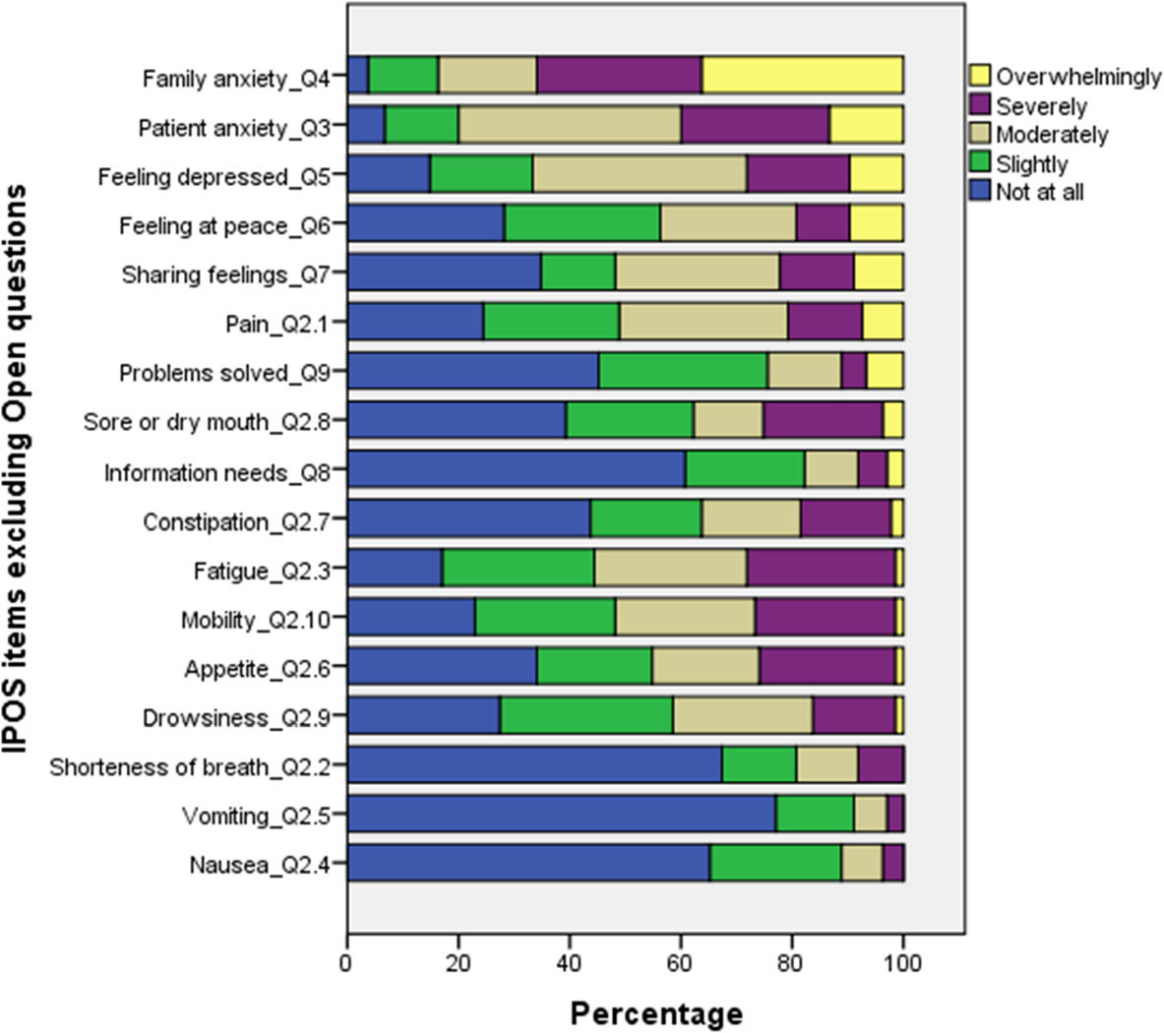
IPOS scores for prevalence of main palliative needs

In terms of prevalence of needs, IPOS items scoring the highest (=4) were: family or friends anxious or worried (36.3%); feeling anxious or worried about illness (13.3%); feeling depressed (9.6%); feeling at peace (9.6%); share feelings (8.9%) and pain (7.4%). IPOS items scoring the lowest (=0) were: vomiting (77%); shortness of breath (67.4%); nausea (65%); information needs (60.7); practical problems (45.2%) and constipation (43%). (See Fig. 1).

### Screening for anxiety and depression

**Fig. 2:**
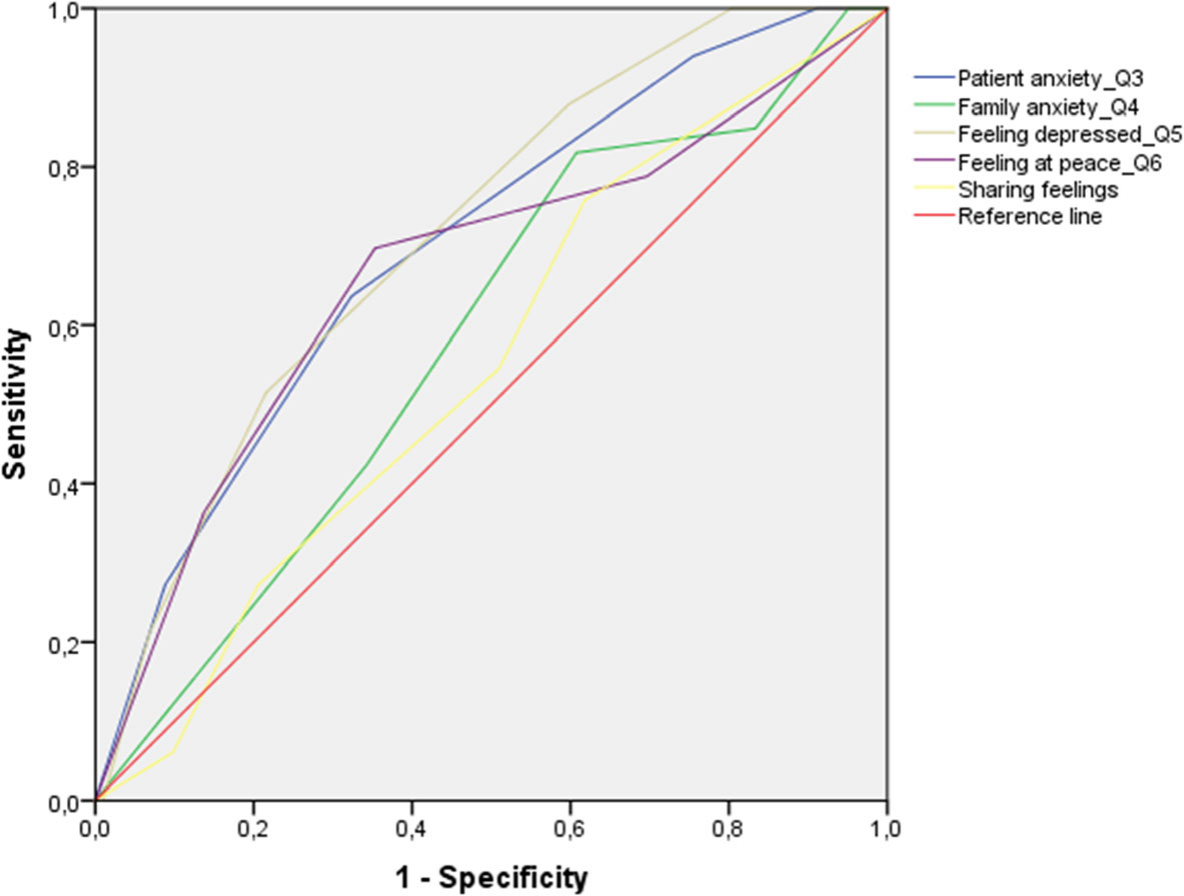
Area Under the Curve for IPOS items 3 to 7

**Fig. 3:**
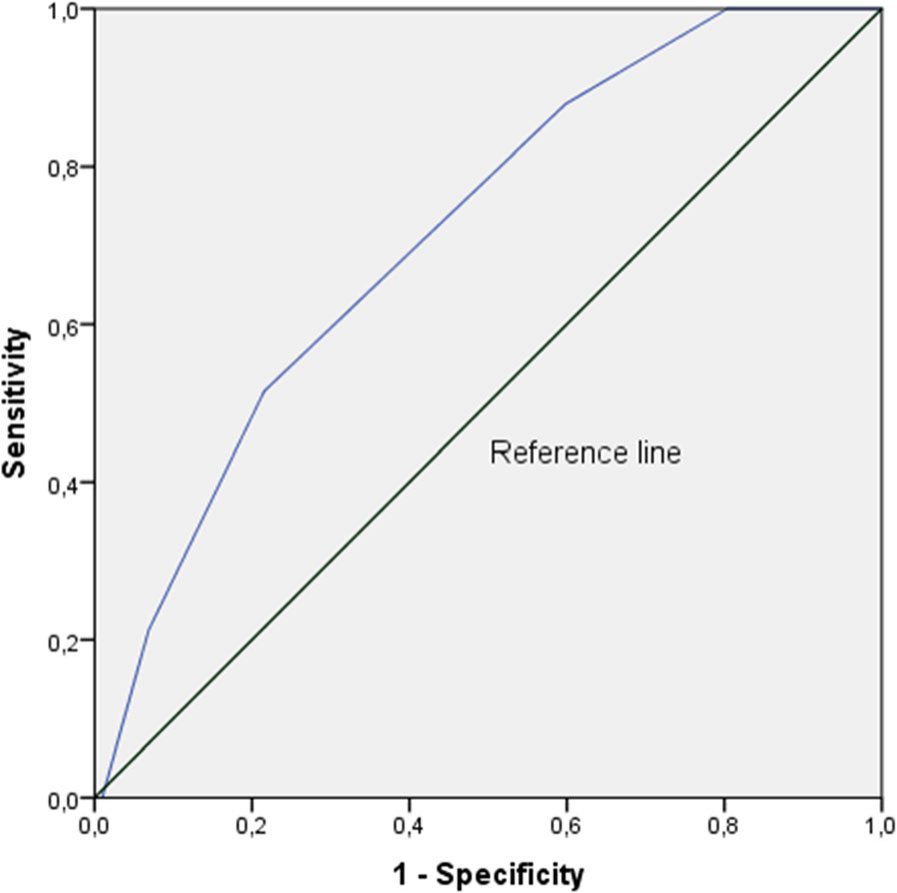
Area Under the Curve for Portuguese IPOS item 5, depression

**Fig. 4:**
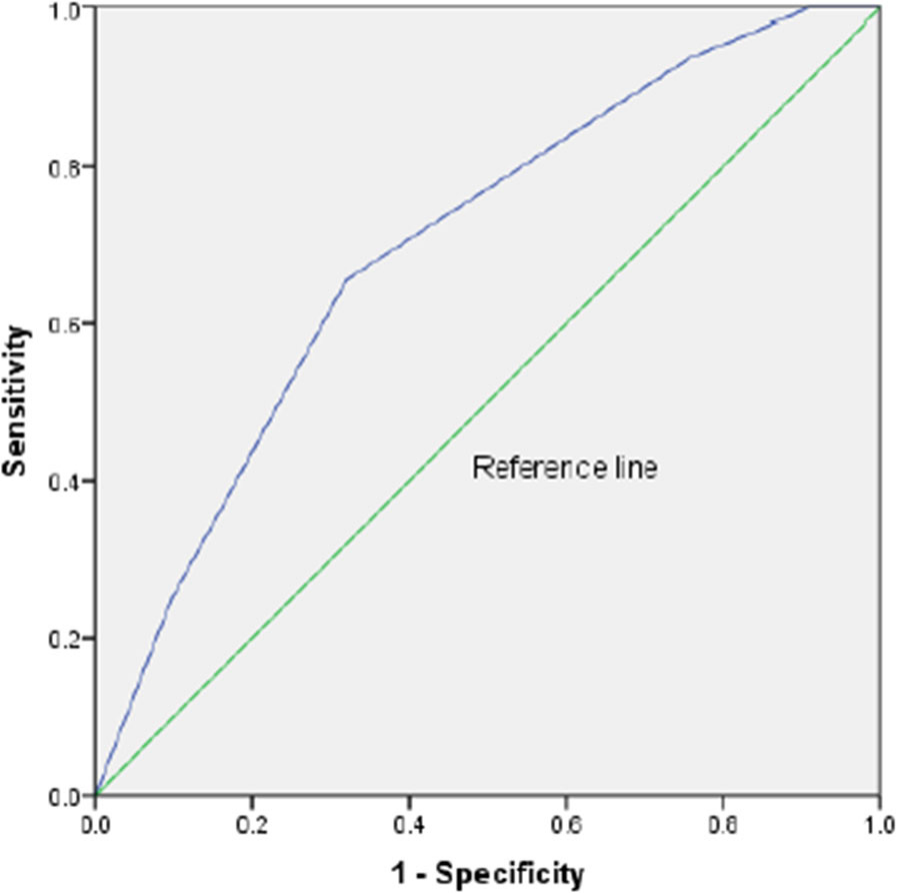
Area Under the Curve for Portuguese IPOS item 3, anxiety

Item 3 (anxiety) and item 5 (depression) presented the highest AUCs (see Fig. 2). The prevalence of depression was 24.4% (C.I. 17.6–32.7%). The AUC curve was 0.72 (C.I.:0.62–0.81), *p* < 0.001 (see Fig. 3). Sensitivity was 51.5% and specificity was 78.4%. Positive predictive value was 43.6% and negative predictive value was 83.3%. As for the anxiety item, the prevalence was 23.7% (C.I. 16.9%–.31.9%) and the AUC was 0.70 (C.I.:0.60–0.80), *p* < 0.001 (see Fig. 4). Sensitivity was 65.6% and specificity was 68.0%. Positive predictive value was 38.8% and negative predictive value was 86.4% (see Table 4).

**Table 4:** - Estimated Values for items 3 and 5, cut-off 2/3, against the HADS subscales, using cut-off 10/11

	Sens (%)	CIs (%)	Spec (%)	CIs (%)	PPV (%)	CIs (%)	NPV (%)	CIs (%)	FPR (%)	CIs (%)	FNR (%)	CIs (%)	Ratios +	CIs (%)	Ratios -	CIs (%)
Item 3 – anxiety Prevalence: 23.7% (C.I. 16.9–31.9%)	65.6	0.47–0.81	68.0	0.58–0.77	38.8	0.26–0.53	86.4	0.77–0.93	0.61	0.47–0.74	0.14	0.07–0.23	0.64	0.43–0.95	0.16	0.09–0.27
Item 5 – depression Prevalence: 24.4% (C.I. 17.6–32.7%)	51.5	0.34–0.69	78.4	0.69–0.86	43.6	0.28–0.60	83.3	0.74–0.90	0.56	0.40–0.72	0.17	0.10–0.26	0.78	0.49–1.21	0.2	0.13–0.31

## Discussion

The main palliative needs of patients cared for by palliative care teams were psychological, family related and spiritual. IPOS systematically identified main needs in this population. Clinical teams seemed to solve or manage physical issues well. This is an extremely positive find. There is evidence that once physical needs are well managed, and the patient is more comfortable, non-physical palliative needs arise [24, 25]. These are also possible to and should be captured systematically in clinical practice, using a patient centred outcome measure and acted upon. In our study, the most stressful non physical issue was family or friends anxious or worried (36.3%). Given that Portuguese culture is based on strong family ties and that decisions are usually made within the family core, this was not surprising. Feeling anxious and depressed were the second and third most stressful issues, even though one of the exclusion criteria to be approached for invitation to participate in the study was being clearly in distress (physical or emotional) judged by direct observation of the healthcare professional. This reinforces the evidence that anxiety is often present in patients with advanced disease due to uncertainties in diagnostic, treatment and prognostic, [13] and, depression is also common among patients with advanced disease [26]. In our sample the prevalence of these issues resonates with Ann-Yi S study in terms of the percentage of patients cared for in the psychology service [8], although that study was conducted only with cancer patients. On the other hand, clusters of physical and non physical symptoms occur and are common in both cancer and non cancer patients [27, 28]. Also, the prevalence of depression in the present study was somewhat higher than the one presented in the Antunes study on screening for depression using one item of the Palliative care Outcome Scale, namely 17.5% (C.I. 14.1–21.6%) [20], but lower than the 30% estimated by Hotopf and colleagues for prevalence of all depressive disorders in advanced disease [29].

Both AUC for anxiety and depression were acceptable (70% or above), although both C.I.s lower levels were slightly below 70%. For cut-off 2/3, both items did not perform well regarding sensitivity, which means these might not be good to identify true positive cases. However, specificity and NPV were good. Both items seem to be good excluding true negative cases, which is one component of screening [30]. These results also reinforce external validity of IPOS.

The main limitation in our study is that the optimal gold standard to screen for anxiety and depression - psychiatric interview for depression as determined by the Diagnostic and Statistical Manual of Mental Disorders (Fifth Edition) was not available to use due to low resources available. The present study used the HADS, a screening measure well accepted in practice and that has been used extensively both in practice and research for several years, nevertheless this is not a diagnostic tool.

Using IPOS systematically could aid clinical teams to track their progress in assisting patients and families with physical and non-physical symptoms. Like other screening tools the Portuguese Integrated Palliative care Outcome Scale seems to be good for excluding true negative cases of depression (item 5) and anxiety (item 3) and can be used to screen patients with advanced disease [30, 31].

## Conclusions

Patient centred outcome measures are powerful communication tools, allowing all those involved in patient care to use a common language, serving not only patients and families, but aiding healthcare professionals, health institutions and policy makers to make evidence supported decisions and improve patient centred care. Building evidence of screening properties of these measures allows not only for patient clinical care, but also to conduct more robust research studies. This study determined screening accuracy properties of the Portuguese Integrated Palliative care Outcome Scale for two psychological related items and shows that this measure can be used to screen patients with advanced disease.

## Data Availability

The datasets generated and/or analysed during the current study are available from the corresponding author on reasonable request.

## Abbreviations

IPOS: Integrated Palliative Outcome Scale
AUC: Area under the curve
HADS: Hospital Anxiety and Depression Scale
ROC: Receiver operating characteristic curve
PPV: Positive predictive value
NPV: Negative predictive value
FNR: False negative rate
FPR: False positive rate
CI: Confidence intervals
